# Functional genomics and microbiome profiling of the Asian longhorned beetle (*Anoplophora glabripennis*) reveal insights into the digestive physiology and nutritional ecology of wood feeding beetles

**DOI:** 10.1186/1471-2164-15-1096

**Published:** 2014-12-12

**Authors:** Erin D Scully, Scott M Geib, John E Carlson, Ming Tien, Duane McKenna, Kelli Hoover

**Affiliations:** Intercollege Program in Genetics at the Huck Institutes of the Life Sciences, The Pennsylvania State University, University Park, Kragujevac, PA 16802 USA; Department of Agronomy and Horticulture, University of Nebraska (UNL)-East Campus and USDA-ARS Grain, Forage, and Bioenergy Research Unit, Lincoln, NE 68583 USA; Tropical Crop and Commodity Protection Research Unit, USDA-ARS Pacific Basin Agricultural Research Center, Hilo, HI 96720 USA; The Schatz Center for Tree Molecular Genetics, Department of Ecosystem Science and Management, The Pennsylvania State University, University Park, Kragujevac, PA 16802 USA; Department of Biochemistry and Molecular Biology, The Pennsylvania State University, University Park, Kragujevac, PA 16802 USA; Department of Biological Sciences, University of Memphis, Memphis, TN 38152 USA; Department of Entomology and Center for Chemical Ecology, The Pennsylvania State University, 501 ASI Building, University Park, Kragujevac, PA 16802 USA

**Keywords:** Metatranscriptome, Cerambycidae, *Fusarium solani*, Nutrient-provisioning, Amino acids, Nitrogen recycling, Xylose fermentation

## Abstract

**Background:**

Wood-feeding beetles harbor an ecologically rich and taxonomically diverse assemblage of gut microbes that appear to promote survival in woody tissue, which is devoid of nitrogen and essential nutrients. Nevertheless, the contributions of these apparent symbionts to digestive physiology and nutritional ecology remain uncharacterized in most beetle lineages.

**Results:**

Through parallel transcriptome profiling of beetle- and microbial- derived mRNAs, we demonstrate that the midgut microbiome of the Asian longhorned beetle (*Anoplophora glabripennis*), a member of the beetle family Cerambycidae, is enriched in biosynthetic pathways for the synthesis of essential amino acids, vitamins, and sterols. Consequently, the midgut microbiome of *A. glabripennis* can provide essential nutrients that the beetle cannot obtain from its woody diet or synthesize itself. The beetle gut microbiota also produce their own suite of transcripts that can enhance lignin degradation, degrade hemicellulose, and ferment xylose and wood sugars. An abundance of cellulases from several glycoside hydrolase families are expressed endogenously by *A. glabripennis*, as well as transcripts that allow the beetle to convert microbe-synthesized essential amino acids into non-essential amino acids*. A. glabripennis* and its gut microbes likely collaborate to digest carbohydrates and convert released sugars and amino acid intermediates into essential nutrients otherwise lacking from their woody host plants.

**Conclusions:**

The nutritional provisioning capabilities of the *A. glabripennis* gut microbiome may contribute to the beetles’ unusually broad host range. The presence of some of the same microbes in the guts of other Cerambycidae and other wood-feeding beetles suggests that partnerships with microbes may be a facilitator of evolutionary radiations in beetles, as in certain other groups of insects, allowing access to novel food sources through enhanced nutritional provisioning.

**Electronic supplementary material:**

The online version of this article (doi:10.1186/1471-2164-15-1096) contains supplementary material, which is available to authorized users.

## Background

Insects are among the most ecologically versatile and taxonomically diverse groups of organisms on the planet, and their symbiotic interactions with microbes are pervasive phenomena [1]. Insect–microbe interactions are known to drive colonization of novel niches, promote survival under harsh environmental conditions [2], provide sources of exogenous genetic material [3], and can facilitate rapid niche expansion and adaptation [4]. The microbial partners of insects include archaea, fungi, protists, and more than 10 phyla of bacteria [5–7]. The taxonomic composition and diversity of insect-associated microbial communities can vary tremendously, even between individuals of the same insect species [8]. Even so, the importance of symbionts to insect nutrition and digestive physiology has been demonstrated repeatedly. For example, symbionts have been shown to manipulate host plant physiology to modulate expression of defense-related genes [9], detoxify ingested plant defensive compounds [10], synthesize essential nutrients [11], fix atmospheric nitrogen, recycle nitrogenous waste products [12], promote host tolerance to extreme environmental conditions [2], digest plant cell wall components [6], and provide protection from pathogenic microbes [13]. Microbial communities associated with insects vary from relatively simple and static intracellular bacterial assemblages [14, 15] to highly complex and dynamic communities comprised of different taxonomic groups of microbes (e.g., fungi and bacteria) such as those associated with wood-feeding (xylophagous) beetles in the family Cerambycidae [7]. In these diverse and complex microbial systems, which are often dominated by facultative symbionts with high degrees of plasticity in terms of community richness and composition [16], it is difficult to determine if and how each of the various microbes directly enhance host fitness. Yet, evidence is mounting that the taxonomic diversity of endosymbionts may be directly correlated with host plant range [17, 18].

Metagenomic approaches using next generation sequencing technologies have successfully elucidated the taxonomic composition and metabolic potentials of a variety of microbial communities associated with phytophagous insects [6, 19–21]. However, one common pitfall of such approaches is that DNA from dead, transient, or metabolically inactive microbes can be sequenced and included in gene annotations, making it difficult to discern between these and microbial taxa that may provide direct fitness benefits to the insect host [22]. Metatranscriptome and/or metaproteome analyses can alleviate these challenges in part, by focusing on microbial taxa and genes that are actively expressed in a particular community and thus potentially contribute to nutritional ecology.

The Asian longhorned beetle (*Anoplophora glabripennis*) belongs to the subfamily Lamiinae in the beetle family Cerambycidae (e.g., cerambycids or longhorned beetles; >30,000 described extant species) [23]. It has an unusually broad host range, attacking and reproducing in at least 47 angiosperm tree species from at least 14 plant families in its native (Asia) and introduced ranges (Europe and North America) [24, 25]. Larvae feed in the sapwood and heartwood of host trees, requiring 1–2 years to complete development, while adult beetles feed on leaves and twigs. *A. glabripennis* is considered to be among the 100 most threatening invasive plant or animal species worldwide [26]. Since the 1980s, *A. glabripennis* has killed millions of trees in China and is responsible for significant losses of high-value shade and timber tree species where it has been introduced in the U.S., Canada, and Europe, with a preference for maples (*Acer spp.*) in its introduced range [24]. Larvae of *A. glabripennis* harbor a complex, diverse, and plastic gut microbiota [17, 20, 27]. Understanding the interactions between *A. glabripennis* and its gut microbial community could lead to new and more effective control practices for this insect and for other xylophagous beetles, in addition to providing new insights into this beetle’s ecology (e.g., symbioses with microbes), evolution, and digestive physiology. For example, control of xylem-feeding insects by insecticides is ineffective [28]; as an alternative the gut microbes that enhance fitness by making key contributions to digestion and nutrient acquisition may be disrupted as a means of biological control as has been previously observed in other wood-feeding insects (e.g., Zootermopsis *angusticollis*) [29].

The larval midgut transcriptome of *A. glabripennis* is rich in insect-derived transcripts predicted to encode cellulases, xylanases, polygalacturonases, detoxification enzymes, and enzymes that could enhance degradation of lignin biopolymers [20, 30]. Even so, functional gaps exist in the beetle digestome, suggesting a role for collaboration with microbes in lignin degradation and the synthesis of essential nutrients [31]. The midgut lumen of *A. glabripennis* is host to a diverse microbial community containing over 300 bacterial and at least 18 fungal operational taxonomic units (OTUs) [20]. Many of the genes detected in the larval midgut metagenome [31] were derived from microbial taxa previously detected in *A. glabripennis* [17, 27, 32, 33], suggesting that a subset of the midgut microbiome is consistently associated with larvae and that enzymes produced by these microbes may play integral roles in digestive physiology. For example, the *A. glabripennis* midgut metagenome [31] contains genes capable of producing a full suite of plant cell wall-degrading enzymes, fixing and recycling nitrogen, synthesizing essential nutrients, detoxifying plant secondary metabolites, and degrading the dominant linkages in hardwood lignin, further demonstrating that the gut microbiome has the metabolic potential to fill functional gaps that exist in the beetle digestome, thereby facilitating survival in woody tissue [20]. Despite these potential contributions to the beetle’s digestive physiology and nutritional ecology, it is not known which gut microbial taxa are metabolically active. Furthermore, although previous studies have characterized the composition of bacterial and fungal communities of the *A. glabripennis* midgut through culture-dependent and culture-independent approaches [17, 27, 31–33], biological replicates were not sampled and it is not known whether any of these microbes are found consistently in the midgut.

The goals of this study were to overcome the limitations of the previous studies by documenting bacterial and fungal taxa that are consistently associated with the midgut of multiple *A. glabripennis* larvae through 16S and ITS amplicon sequencing (“microbiome profiling”). These persistant microbes may have deep evolutionary relationships with *A. glabripennis* and we hypothesize that these microbes are primed to serve key roles in digestion and nutrient provisioning. Additionally, metatranscriptome profiling of the midgut microbiome was performed to identify transcriptionally active microbes in the midgut and to characterize their metabolic potential. Using these methods, we are able to provide an overview of the microbial gene expression profile in actively-feeding larvae and detail the relative contributions of these microbes to digestive and physiological processes. We compared the functions of the genes expressed by the gut microbiota to those endogenously expressed by *A. glabripennis* midgut in order to identify gaps in insect digestive physiology that can be complemented by genes expressed by gut symbionts. Taken together, this study reveals important new insights into *A. glabripennis’* digestive physiology and nutritional biology, which will ultimately facilitate further studies of the ecology, physiological relevance and evolutionary history of symbioses between xylophagous beetles and microbes.

## Results and discussion

### Midgut bacterial community structure inferred from 16S rDNA amplicon sequencing

**Figure 1:**
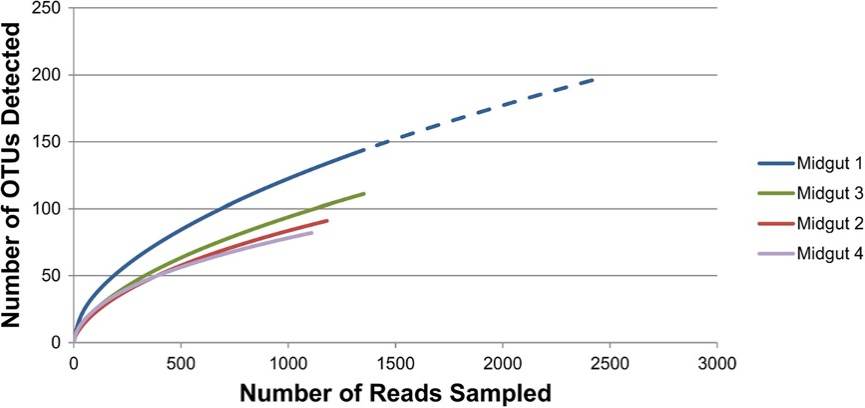
**Rarefaction analysis of 16S amplicons sequenced from four individual third instar larvae of** ***A. glabripennis*** **feeding on sugar maple.** Curves failed to reach saturation, indicating that the community may harbor additional OTUs not sampled for sequencing. This hypothesis is supported by 20 additional OTUs detected by further sequencing of Sample 1 (dotted line).

**Figure 2:**
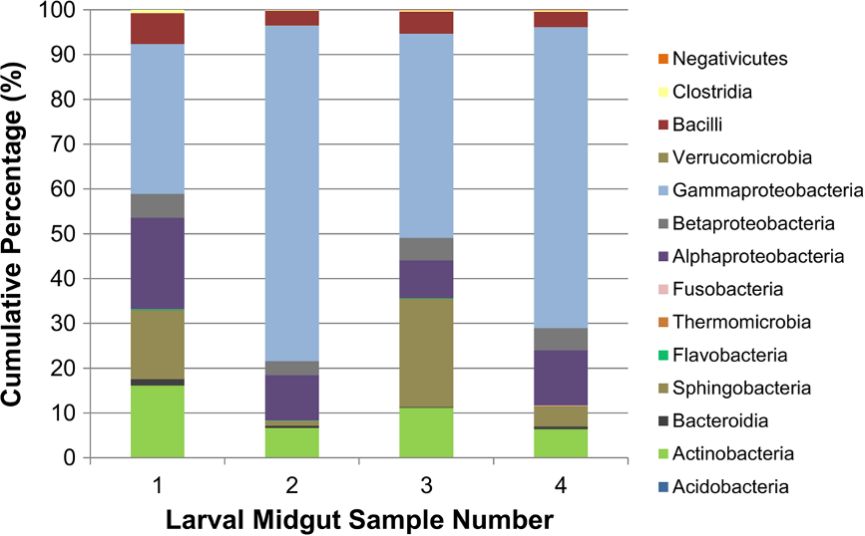
**Relative abundances of bacterial classes detected through 16S amplicon analysis of four individual third instar larvae of** ***A. glabripennis*** **.**

Species richness varied slightly among individual larval midgut communities, ranging from 82 to 198 operational taxonomic units (OTUs) at a 97% sequence similarity threshold. Combined richness for all bacterial midgut communities sampled was 317 OTUs. Measures of community complexity and diversity varied slightly among individuals (Table 1). Rarefaction curves computed for each individual midgut community failed to reach saturation (Figure 1), suggesting that the full richness of the individual midgut communities was not entirely sampled by sequencing. The midgut bacterial communities were predominately composed of members of the following classes: Actinobacteria, Alphaproteobacteria, Bacilli, Bacteroidetes, Clostridia, Gammaproteobacteria, and Sphingobacteria (Figure 2). The communities were generally dominated by OTUs assigned to class Gammaproteobacteria and contained approximately equivalent relative abundances of OTUs classified to Bacilli and Betaproteobacteria, while the relative abundances of Actinobacteria, Alphaproteobacteria, Gammaproteobacteria, and Sphingobacteria differed slightly among individual beetles. Twenty-two OTUs were detected in association with all midgut communities sampled (Additional file 1: Table S1), while two OTUs assigned to the family Enterobacteriaceae were highly abundant in all midguts sampled for sequencing. These could not be definitively classified to the genus level, but had highest scoring BLASTN alignments to bacteria in the genus *Enterobacter* in the RDP classifier database. Interestingly, unclassified members of the family Enterobacteriaceae were consistently associated with the egg, oviposition site, and *A. glabripennis* larvae in a previous study and were flagged as candidates for vertical transmission, suggesting an intimate association with the larval stage of this insect [34].

**Table 1:** **Ecological indices for 16S bacterial communities sampled from the midguts of each of 4 individual** ***A. glabripennis*** **larvae feeding in sugar maple**

Sample	Number OTUs	Chao	Chao 95% CI	Ace	Ace 95% CI	Shannon	Shannon 95% CI	Simpson	Simpson 95% CI
1	198	451	342–641	659	565–776	3.69	3.62–3.74	0.043	0.041–0.046
2	91	156	121–230	361	199–315	2.40	2.29–2.50	0.200	0.185–0.217
3	111	246	178–385	200	295–451	2.90	2.81–2.98	0.100	0.093–0.107
4	82	141	107–218	142	84–287	2.80	2.70–2.99	0.115	0.106–0.125

The midgut microbial communities of larval *A. glabripennis* sampled herein were relatively distinct from those sampled in previous studies from larval *A. glabripennis* collected in Worcester, MA (introduced range) [20] or from China (native range) [17]; instead the larvae sampled for this study more strongly resembled microbial communities from *A. glabripennis* larvae collected in Brooklyn, NY [27]. The bacterial communities of larval *A. glabripennis* from Worcester, MA and China were dominated by Bacilli [17, 20] while those sampled from New York were predominately assigned to class Gammaproteobacteria [27]. The differences in the taxonomic compositions of these communities are not surprising since the Worcester, MA and New York populations were introduced separately and originated from different source populations in Asia [35]. Additionally, our *A. glabripennis* colony was derived primarily from the New York population and, therefore, the resemblance of the midgut bacterial communities was expected.

Because a different region of the 16S rRNA locus was targeted for sequencing in the prior studies referenced in the above paragraph, direct OTU comparisons cannot be performed. However, several genera were detected in both communities, including *Aeromicrobium*, *Aurantimonas, Agrococcus, Brachybacterium, Brevibacterium*, *Chrysobacterium, Curtobacterium*, *Enterococcus, Flavobacterium*, *Leucobacter*, *Microbacterium*, *Olivibacter*, *Pseudomonas*, *Pseudonocardia*, *Sphingobacterium*, *Staphylococcus, Stenotrophomonas*, and *Streptococcus*. Further, OTUs assigned to the genus *Pseudomonas* were highly abundant in all three populations (i.e., New York, Massachusetts, and the Penn State University (PSU) colony).

### Midgut fungal community structure inferred from ITS amplicon sequencing

**Figure 3:**
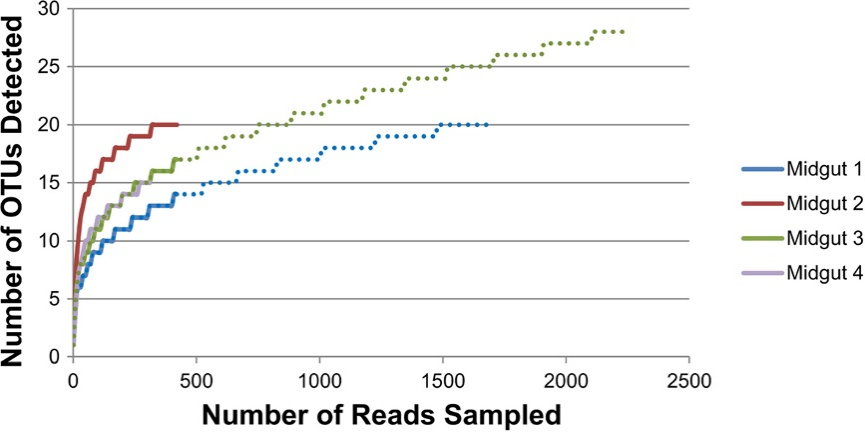
**Rarefaction analysis of ITS fungal amplicons sequenced from the midguts of four individual third instar larvae of** ***A. glabripennis*** **.** Rarefaction curves appeared to reach saturation, indicating sufficient sampling to detect the majority of the fungal community diversity, although deeper sampling of samples 1 and 3 revealed the detection of 6 and 12 additional OTUs, respectively (dotted line).

Observed richness values ranged from 15 to 28 OTUs, which is in agreement with the fungal community richness detected previously through 18S amplicon sequencing [20]. Total richness among all midgut fungal communities sampled was 44 OTUs (Table 2). Rarefaction curves approached saturation and computed values for various community richness estimators were similar to the number of observed OTUs in communities from each individual beetle midgut sampled, indicating adequate sampling (Figure 3). The Boneh estimator, which predicts the detection of additional OTUs with subsequent sampling, projected detection of 1–10 additional OTUs, supporting the hypothesis that the sequencing depth achieved for the fungal ITS communities was adequate to detect the majority of the fungal OTUs associated with the *A. glabripennis* larval midgut.

**Table 2:** **Ecological indices for ITS fungal communities sampled from the midguts of each of four individual third instar larvae of** ***A. glabripennis*** **feeding in the heartwood of sugar maple (** ***Acer saccharum*** **)**

Sample	Number OTUs	Chao	Chao 95% CI	Ace	Ace 95% CI	Shannon	Shannon 95% CI	Simpson	Simpson 95% CI
1	20	23	20–37	25	21–45	1.74	1.70–1.79	0.22	0.21–0.23
2	20	20	20–26	22	20–33	2.52	2.44–2.60	0.10	0.09–0.11
3	28	43	32–92	83	57–134	1.89	1.85–1.94	0.22	0.21–0.23
4	15	21	16–53	22	17–39	1.67	1.52–1.82	0.33	0.28–0.39

**Figure 4:**
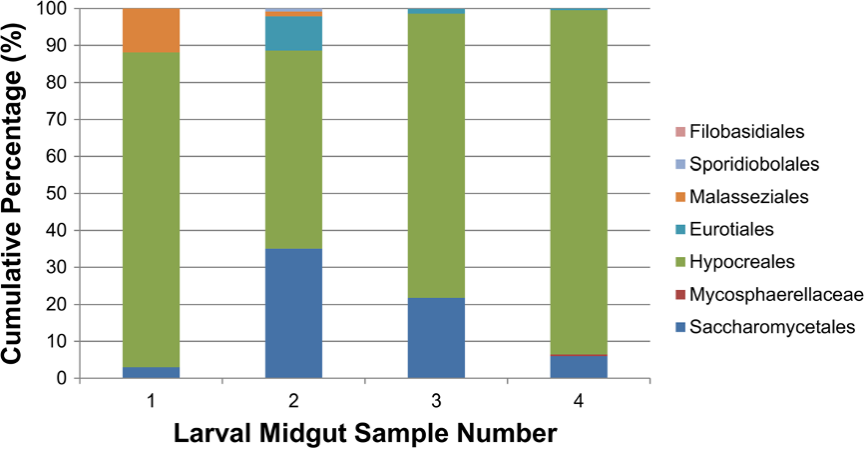
**Abundance of fungal orders detected in ITS amplicon data sampled from the midguts of four individual third instar larvae of** ***A. glabripennis*** **.** Fungal reads were exclusively classified to phylum Ascomycota. At the ordinal level, the communities were dominated by Hypocreales and Saccharomycetales.

**Figure 5:**
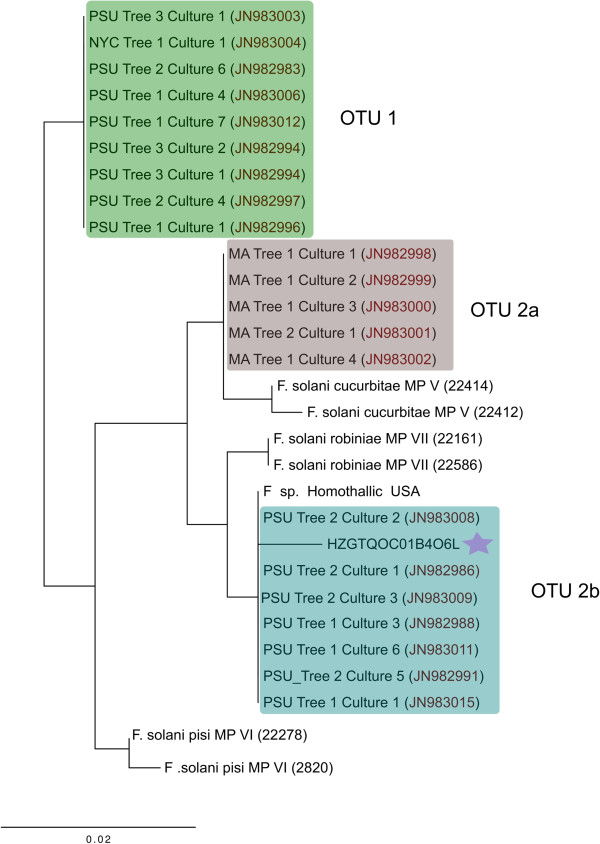
**Maximum likelihood analysis of fungal ITS amplicon sequences taxonomically assigned to** ***Fusarium solani*** **that were detected in** ***A. glabripennis*** **larval midguts.** The star designates the *F. solani* derived OTU that was detected in the current study and scale bars represent the number of substitutions per site. PSU: *F. solani* isolates cultivated from *A. glabripennis* larvae reared at Penn State University; MA: represents *F. solani* cultivated from *A. glabripennis* larvae collected from a field site in Worcester, MA; NYC represents *F. solani* isolated from *A. glabripennis* larvae collected from a field site in Brooklyn, NY. MP designates the *F. solani* mating population number.

The midgut fungal community was more stable than the bacterial community and contained a greater percentage of OTUs that were detected in all beetle gut microbiota sampled in this study. For example, gut communities were consistently dominated by ITS amplicons assigned to the order Hypocreales (Figure 4). Seven OTUs, including six assigned to the genus *Fusarium* and a single OTU assigned to the genus *Pichia*, were associated with all *A. glabripennis* larval midguts sampled in this study. Fungi belonging to the *Fusarium solani* species complex 6 (FSSC6) have been consistently detected in the midgut of several populations of *A. glabripennis* sampled for sequencing [33]. Further, one of the OTUs detected in this analysis was 98% similar at the nucleotide sequence level to a *Fusarium solani* OTU detected previously from beetles sampled from the PSU *A. glabripennis* colony [33], indicating persistent maintenance of these OTUs over multiple generations, likely through vertical transmission. Further study is necessary to elucidate whether the consistent association of *F. solani* with *A. glabripennis* signifies the importance of this fungus to the beetle’s fitness and digestive physiology. Phylogenetic analysis under maximum likelihood inference illustrates that this OTU is more closely related to an isolate detected in the PSU colony than *Fusarium*-derived OTUs isolated from *A. glabripennis* midguts from the Worcester, MA population (Figure 5) [33]. Six OTUs with highest scoring BLASTN alignments to *Fusarium oxysporum* were also detected through this analysis, which was detected previously in the *A. glabripennis* midgut (unpublished data)*.*

### Assembly and annotation metrics

Over 65% of the Illumina paired end reads resulting from sequencing the midgut contents library and over 95% of the reads from the intact midgut library passed quality filtering. Using Trinity, 161,177 transcript isoforms from 97,506 genes ranging in length from 200 nt to 31,393 nt (N50 contig length: 684 nt) were assembled from the midgut contents library, while 61,812 transcript isoforms from 45,418 genes ranging in length from 200 nt to 26,118 nt (N50 contig length: 592) were assembled from the intact midgut library (Table 3).

**Table 3:** **Trinity assembly metrics for** ***A. glabripennis*** **transcripts obtained separately from midgut contents and from intact midgut transcriptome libraries (see** Methods for more information)

Source	Number of transcripts	Minimum transcript length (nt)	N80 transcript length (nt)	N50 transcript length (nt)	N20 transcript length (nt)	Maximum transcript length (nt)	Total length of assembled transcripts (nt)
**Midgut contents**	161,117	200	323	684	1,945	31,383	90.09 Mb
**Midgut contents: Microbial**	**7,952**	**200**	**245**	**334**	**546**	**5,049**	**2.69 Mb**
**Midgut contents: Bacterial**	3,084	200	247	363	653	5,049	1.12 Mb
**Midgut contents: Fungal**	4,868	200	242	316	484	2,259	1.58 Mb
**Intact midgut**	61,812	200	272	592	1,937	26,118	30.6 Mb
**Intact midgut: Microbial**	**3,167**	**200**	**266**	**492**	**1,271**	**14,152**	**1.42 Mb**
**Intact midgut: Bacterial**	2,154	200	294	548	1,445	14,152	1.05 Mb
**Intact midgut: Fungal**	1,013	200	234	353	799	3,643	0.36 Mb

**Figure 6:**
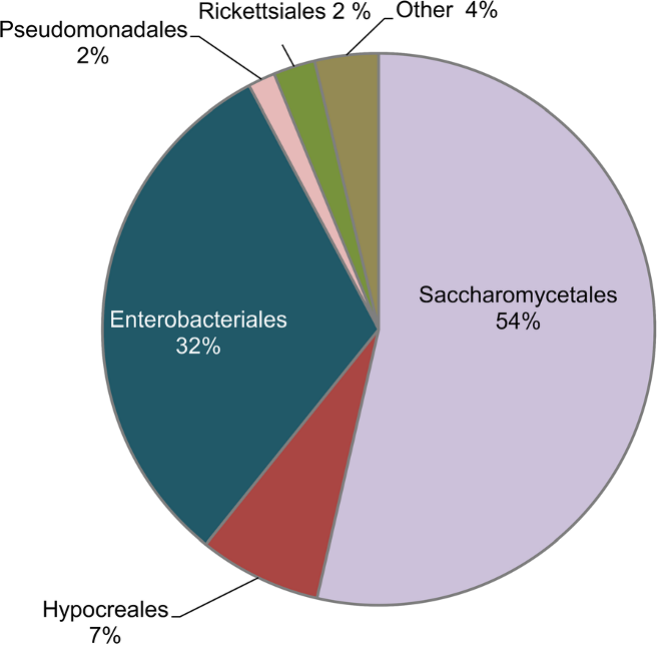
**Bacterial and fungal classes detected in** ***A. glabripennis*** **larval intact midgut and midgut contents metatranscriptome assemblies through MEGAN analysis of putative protein coding genes.**

Of the assembled transcripts, 7,952 (2.7 Mb) and 3,167 (1.42 Mb) were identified as microbial from the midgut contents and intact midgut libraries, respectively, and had highest scoring BLASTX alignments to microbial protein coding genes present in the NCBI non-redundant protein database at an e-value of 1e-5 or lower, a minimum alignment length of 15 amino acids, and at least a 60% amino acid similarity (Table 4). Relative expression levels of microbial-derived functional gene categories that are relevant for survival in woody tissue are presented in Table 5. Transcripts in the midgut library were assigned to six bacterial and eight fungal orders, while transcripts in the midgut contents library were assigned to 14 bacterial and five fungal orders (Figure 6). In both libraries, Saccharomycetales was the dominant fungal order, while the bacterial orders Enterobacteriales and Lactobacillales were dominant in the midgut contents and intact midgut libraries, respectively (Additional file 2: Figure S1).

**Table 4:** **Annotation statistics for microbial transcripts detected in the** ***A. glabripennis*** **midgut contents and intact midgut libraries**

	Midgut contents	Intact midgut
Number of rRNAs	182	237
Number of Transcripts with BLASTX Alignments	7,952	3,167
Number of Transcripts with Gene Ontology Assignments	1,083	1,225
Number of Transcripts with KEGG Assignments	705	1,103
Number of Transcripts with Pfam Assignments	1,686	2,766
Number of Transcripts with KOG/COG assignments	1,554	2,328

**Table 5:** **Number of transcripts per million mapped reads (TPM) for bacterial and fungal transcripts assembled from the midgut contents and intact midgut libraries**

Class	COG assignment	TPM midgut contents	TPM intact midgut
Actinobacteria	Amino acid transport and metabolism	0	0.72
	Carbohydrate transport and metabolism	7.43	2.04
	Cell motility and secretion	0	1.08
	Coenzyme metabolism	0	1.28
	Energy production and conversion	0	2.24
	Function unknown	0	2.67
	Lipid metabolism	0	1.08
	Nucleotide transport and metabolism	0	2.01
Alphaproteobacteria	Amino acid transport and metabolism	3.28	0
	Cell envelope biogenesis, outer membrane	6.47	0
	Coenzyme metabolism	3.03	0
	Energy production and conversion	5.04	0
	General function prediction only	10.17	0
Bacilli	Amino acid transport and metabolism	0	326.88
	Carbohydrate transport and metabolism	558.13	492.9
	Cell envelope biogenesis, outer membrane	1585.16	334.75
	Cell motility and secretion	1569.88	41.89
	Coenzyme metabolism	0	157
	Energy production and conversion	0	232.11
	General function prediction only	30.88	568.52
	Inorganic ion transport and metabolism	0	257.6
	Lipid metabolism	0	95.11
	Nucleotide transport and metabolism	0	214.12
	Secondary metabolites biosynthesis, transport, and catabolism	0	86.14
Bacteroidetes	Energy production and conversion	1.51	0
	Function unknown	3.47	0
Betaproteobacteria	Coenzyme metabolism	1.94	0
	Cell envelope biogenesis, outer membrane	21.71	0
Clostridia	Function unknown	342.46	0
Gammaproteobacteria	Amino acid transport and metabolism	105.84	1.11
	Carbohydrate transport and metabolism	147.84	0.33
	Cell envelope biogenesis, outer membrane	304.28	1.96
	Cell motility and secretion	111.13	0
	Coenzyme metabolism	49.54	0
	Defense mechanisms	20.49	1.36
	Energy production and conversion	168.14	1.17
	Function unknown	189.35	0.24
	General function prediction only	187.36	1.94
	Inorganic ion transport and metabolism	185.12	0
	Lipid metabolism	15.69	0.23
	Nucleotide transport and metabolism	81.51	0
Saccharomycetes	Amino acid transport and metabolism	172.97	0
	Carbohydrate transport and metabolism	106.88	0.33
	Cell motility	5.21	0
	Cell wall/membrane/envelope biogenesis	107.45	0
	Chromatin structure and dynamics	32.59	0
	Coenzyme transport and metabolism	17.03	0
	Defense mechanisms	98.79	0
	Energy production and conversion	64.95	0
	Extracellular structures	93.48	0
	Function unknown	156.44	0
	General function prediction only	348.77	0
	Inorganic ion transport and metabolism	193.37	0
	Lipid transport and metabolism	85.25	0
	Nucleotide transport and metabolism	34.03	0.96
Sordariomycetes	Amino acid transport and metabolism	10.10	0
	Carbohydrate transport and metabolism	7.40	0
	Cell wall/membrane/envelope biogenesis	1.47	0
	Chromatin structure and dynamics	5.63	0
	Coenzyme transport and metabolism	1.02	0
	Energy production and conversion	8.14	0
	Function unknown	0.56	2.26
	General function prediction only	14.59	0
	Inorganic ion transport and metabolism	8.62	0
	Lipid transport and metabolism	1.47	0
	Nucleotide transport and metabolism	3.35	0
	Secondary metabolites biosynthesis, transport and catabolism	1.65	0

Class-level taxonomic assignments and COG (Clusters of Orthologous Genes) functional assignments are indicated.

### Bacterial 16S and fungal ITS OTUs detected in metatranscriptome data

Over 500 transcripts were classified as SSU (small ribosomal subunit) or LSU (large ribosomal subunit) rRNAs (Additional file 3: Table S2). The expression of several persistent OTUs in all beetle midgut communities sampled for sequencing was confirmed. For example, rRNAs with highest scoring BLASTN alignments to eight shared OTUs were detected and included OTUs classified to the genera *Novosphingobium*, *Propionibacterium*, *Pseudomonas*, *Pediococcus*, and *Staphylococcus*; the families Burkholderiaceae and Enterobacteriaceae (2 OTUs); and the order Actinomycetales. However, the majority of the identified rRNAs were from OTUs detected in association with the midgut communities of just one or two of the larvae sampled (Table 6). Interestingly, the majority of the 16S rRNA transcripts constructed from the Illumina reads did not have significant BLASTN matches to OTUs documented through community profiling. In some cases, the transcripts originated from a different region of the 16S rRNA locus than the region targeted for amplicon sequencing and therefore would not be expected to align with the OTUs detected in the amplicon analysis. However, in many cases, the transcripts overlapped the region targeted for amplicon analysis, but had low sequence identities to the 16S OTUs. This could be a reflection of the dynamic nature of the midgut community, as has been observed for the gut communities of other xylophagous insects [36], or it could be an artifact of feeding on a non-sterile food source where DNA from microbes ingested during feeding is more abundant than DNA from microbes that are truly residents of the midgut. Consistent with the utility of targeting expressed rRNAs for symbiont community analysis, the taxonomic classification of the rRNAs that were assembled from the transcriptome libraries most strongly resembled the taxonomic assignments obtained from MEGAN analysis of the microbial protein coding genes.

**Table 6:** **Taxonomic identity of 16S OTUs supported by rRNAs assembled from metatranscriptome data for intact midgut and midgut contents**

Classification to lowest possible taxonomic rank	Percent nucleotide identity	Number of assembled rRNAs	Persistant?
Acinetobacter	97	1	No
Actinomyces	100	1	No
Actinomycetales	95	2	No
Actinomycetales	96	2	Yes
Actinomycetales	96	1	No
Bacteria	96	1	No
Burkholderiaceae	97	1	Yes
Caryophanon	100	1	No
Cellovibrio	98	2	No
Curtobacterium	97	1	No
Enterobacteriaceae	99	2	No
Enterobacteriaceae	97	3	No
Enterobacteriaceae	96	4	No
Enterobacteriaceae	98	1	Yes
Enterobacteriaceae	95	1	Yes
Novosphingobium	96	1	Yes
Pasteurellaceae	95	1	No
Pediococcus	95	1	Yes
Propionibacterium	99	1	Yes
Pseudomonadaceae	100	1	No
Pseudomonadaceae	97	1	No
Pseudomonas	97	1	Yes
Sphingobacterium	98	1	No
Staphylococcus	99	1	Yes
Streptococcus	98	1	No

Ribosomal RNAs assembled from the intact midgut and midgut contents metatranscriptome data were compared to the OTUs detected in the 16S amplicon data using BLASTN to determine which OTUs were transcriptionally active in the midgut. 16S OTUs were considered persistant if they were detected in ≥ 3 *A. glabripennis* midgut communities in the current study.

Although fungal ITS sequences were not numerous in the metatranscriptome data (the ITS region is generally excised from mature rRNAs) they can be present in pre-ribosomal RNAs. Five complete ITS transcripts were identified in both the intact midgut and midgut contents. Two of these transcripts had 100% nucleotide identity to persistent OTUs detected in all midgut communities and both were classified to the genus *Pichia*. While transcriptional activities of other fungal OTUs could not be confirmed, analysis of SSUs and LSUs detected in the metatranscriptome data confirmed that many of the persistent fungal OTUs identified through ITS community profiling were transcriptionally active (e.g., *Fusarium* and *Candida*).

### Metabolic capacity of the *A. glabripennis*midgut microbiome

**Figure 7:**
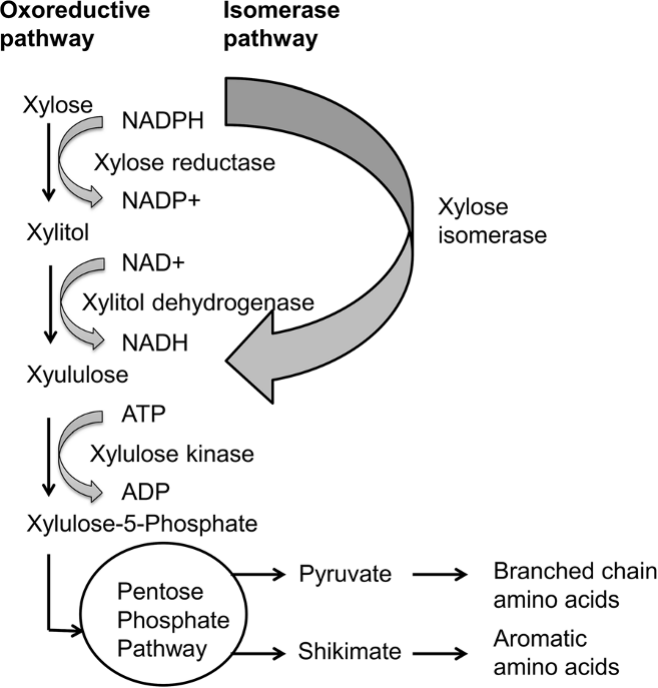
**Putative pathways for xylose utilization based on BLASTX annotation of transcripts sampled from the midgut microbiota of larval** ***A. glabripennis*** **.** Xylose can be shuttled into the pentose phosphate pathway by two different routes detected in the metatranscriptome: the oxoreductive pathway and the isomerase pathway. Transcripts originating from the oxoreductive pathway were from yeasts while transcripts originating from the isomerase pathway were from lactic acid bacteria. Both pathways lead to the production of xylulose-5-phosphate, which is shuttled into the pentose phosphate pathway and is used to produce pyruvate and shikimate. These compounds serve as key intermediates in the synthesis of essential branched chain amino acids and essential aromatic amino acids, demonstrating how these wood sugars can be used by the gut microbiota to produce essential nutrients that are otherwise lacking from the *A. glabripennis* diet.

The midgut microbiome comprised a much broader repertoire of genes with predicted involvement in carbohydrate digestion and sugar assimilation relative to the *A. glabripennis* midgut transcriptome (Additional file 4: Table S3). Many of these microbial genes could potentially complement *A. glabripennis’* existing repertiore of digestive and nutrient assimilating capacities. This capacity was noted particularly with regard to utilization of hemicellulosic sugars. For example, insect-derived xylanases, which are hypothesized to release xylose sugars from the xylan chains that predominantly comprise hemicellulose found in angiospermous trees, have been documented in the *A. glabripennis* midgut through both transcriptome and zymogram analyses [31, 37]. However, pathways for xylose sugar metabolism and utilization were not detected in the *A. glabripennis* transcriptome [31] and have not been identified in any other insect. The microbial community of *A. glabripennis* actively expressed genes with predicted roles in the assimilation and metabolism of xylose sugars, which could be used for energy production or as substrates for various biosynthetic pathways (i.e., carbon skeletons of amino acids or fatty acids). One of the most striking discoveries was the expression of xylose sugar utilization pathways by the fungal microbiota via the oxido-reductase pathway, which is a pathway used to convert xylose to D-xylulose-5-phosphate that can then enter the pentose phosphate pathway (Figure 7). In addition, we found lactic acid bacterial-derived transcripts (e.g. *Pediococcus*) from the xylose isomerase pathway that are capable of converting xylose directly into D-xylulose. It has been previously hypothesized that xylose-fermenting yeasts commonly found in the guts of cerambycid beetles play roles in processing xylose [38, 39], converting it into metabolites that could be used by the host or the microbiota in various biosynthetic processes. The expression of yeast-derived oxido-reductase and bacterial-derived xylose isomerase pathways in *A. glabripennis* supports a role for the gut microbiota in xylose sugar utilization. Whether these microbes are simply exploiting xylose sugars released from hemicellulose by beetle-derived xylanases or are actually working “in cooperation” with *A. glabripennis* to digest hemicellulose and utilize its metabolites is not known.

Fungal transcripts predicted to encode glycoside hydrolase family 28 (GH 28) polygalacturonases and GH 5 cellulases were also expressed in the *A. glabripennis* midgut, indicating that the fungal community is capable of degrading cellulose and other plant polysaccharides in woody tissue (e.g., pectin). Fungal and bacterial transcripts predicted to encode ABC transporters, β-glucosidases, β-xylosidases, major facilitator superfamily transporters, and phosphotransferase systems, were detected, suggesting that microbes are also capable of assimilating and metabolizing sugars released from secondary cell wall polysaccharides. Of particular interest are the transcripts predicted to encode components of phosphotransferase systems since many of these can internalize and metabolize cellobiose, xylose, mannose, galactose, and other sugars released from woody tissue by beetle-derived cellulases and hemicellulases. While *A. glabripennis* can endogenously metabolize and use many of the hexose sugar substrates for energy production, members of the microbiota expressed transcripts that could ferment both 5- and 6- carbon sugars, allowing hexoses and pentoses liberated from the plant cell wall to be converted into substrates that can be used in various anabolic processes, i.e., amino acid or fatty acid biosynthesis.

**Figure 8:**
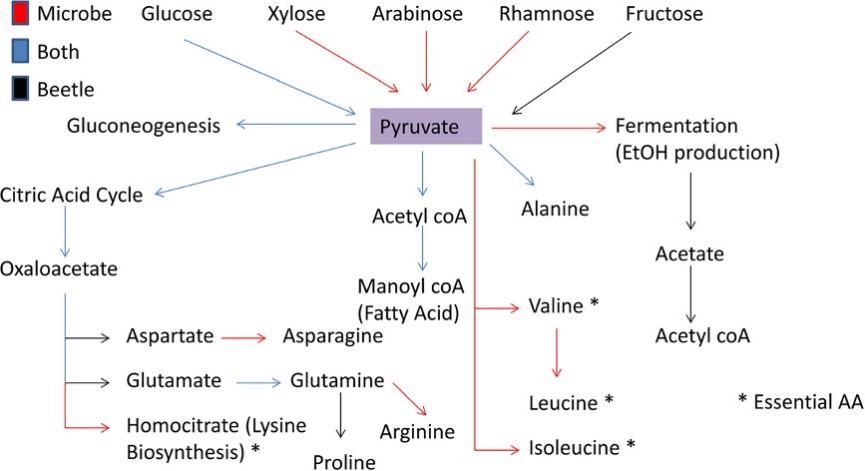
**Pathways for pyruvate utilization detected in the** ***A. glabripennis*** **larval midgut microbiome.** The gut microbial community has an expanded capacity to synthesize pyruvate from pentose sugars found in hemicellulose and convert pyruvate to essential branched-chain amino acids and homocitrate, an essential component of the lysine biosynthetic pathway.

**Figure 9:**
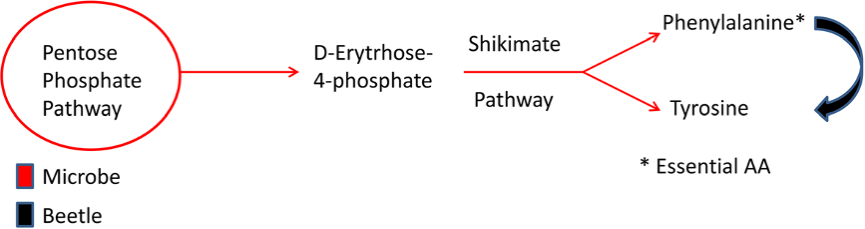
**Partial pathways for aromatic amino acid biosynthesis detected in the** ***A. glabripennis*** **larval midgut microbiome.** In some cases, pathways encoded by the gut microbiota can be complemented by transcripts derived from *A. glabripennis*. For example, gut microbes encoded full pathways for the biosynthesis of the essential aromatic amino acid phenylalanine, while *A. glabripennis* produces the enzymes necessary to convert phenylalanine derived from microbes into tyrosine.

Based on our data, the gut microbial community likely benefits *A. glabripennis* by *i)* increasing it’s capacity to utilize 5-carbon sugars and *ii)* by providing biosynthetic pathways for essential biosynthetic precursors (Figure 8). For example, while the beetle can metabolize glucose and fructose into pyruvate, the gut microbiota can convert many other sugar substrates, including pentose sugars found in hemicellulose from angiospermous trees into this key metabolic intermediate. This is a potential mechanism for the conversion of glucose and xylose released from plant cell walls by beetle digestive enzymes into essential nutrients by the gut community. Microbial pathways for the conversion of pyruvate into essential branched chain amino acids (valine/leucine/isoleucine) and homocitrate, which is a valuable substrate for lysine biosynthesis (another essential amino acid), were expressed in the midgut (Figure 9). Although additional experimental evidence is needed to validate the microbial conversion of wood sugars into essential nutrients, biochemical evidence using ^13^C labeled carbon suggests a microbial origin of the carbon backbones of several essential amino acids in the *A. glabripennis* midgut (Ayayee, in review).

Aside from its potential utility in the biosynthesis of essential and nonessential amino acids, pyruvate can also be used to produce substrates for fatty acid biosynthesis. For example, the gut microbiota can synthesize acetyl CoA and malonyl CoA from pyruvate, which are required for biosynthesis of fatty acids and are scarce in woody tissue, via a transcriptionally active pentose phosphate pathway capable of converting five-carbon sugars (e.g. xylose and arabinose) into intermediates that can be used in biosynthesis of essential aromatic amino acids by the shikimate pathway. Full pathways for phenylalanine biosynthesis and partial pathways for histidine acid biosynthesis were detected in the microbial-derived transcripts. Although we did not find pathways in the gut involved in biosynthesis of tryptophan in either the metatranscriptome or the larval transcriptome, *A. glabripennis* produces transcripts predicted to encode enzymes necessary to convert phenylalanine to tyrosine. The microbial origin several of essential aromatic and branched chain amino acids in *A. glabripennis* was validated by ^15^ N enrichment experiments [12]. Although the beetle is capable of synthesizing several non-essential amino acids from pyruvate [31], the microbiota also expressed components of pathways for the synthesis of several non-essential amino acids, such as asparagine, glutamate, glutamine, and arginine. The synthesis of non-essential amino acids by gut microbes may contribute to nitrogen conservation in *A. glabripennis.*

The gut microbiota can also make contributions to the nitrogen economy in the midgut by recycling nitrogenous waste products produced either by the beetle or gut microbes, which is consistent with previous work showing that nitrogen recycled from urea is reincorporated into both essential and non-essential amino acids in larval *A. glabripennis* [12]. However, alternate pathways for recycling nitrogenous waste products were also detected. For example, transcripts predicted to encode NAD-specific glutamate dehydrogenases, taurine dehydrogenases, and glutamine synthetases were expressed by various members of the gut community. Glutamate dehydrogenase typically converts glutamate to α-ketogluturate for excretion; however, it can also catalyze the reverse reaction under ammonia-rich conditions, recycling α-ketoglutarate waste products back into glutamate. Glutamine synthetases can also have integral roles in nitrogen economy by incorporating ammonia liberated from amino acid and nucleic acid deamination reactions back into glutamine. Taurine dioxygenases may also contribute to nitrogen economy in the gut because these enzymes can actively break down the organic acid taurine, which is one of the most prominent free organic acids in both insect hemolymph and tissues [40]. Taurine dioxygenases catalyze the conversion of taurine to sulfite, aminoacetaldehyde, carbon dioxide, and succinate, allowing it to be used as a source of both nitrogen and sulfur. Finally, transcripts containing xanthine uracil permease domains and phosphoribosyltransferase domains were found, which participate in the purine salvage pathway and signify a potential microbial role for the recycling of purine nucleotides.

### Potential opportunities for nutrient exchange between microbes and *A. glabripennis*

We found numerous opportunities for nutrient and metabolite exchange between members of the midgut microbiota and *A. glabripennis,* which could contribute to niche expansion by *A. glabripennis,* represented by its ability to colonize and thrive in an unusually broad range of healthy host trees. For example, potential pathway complementarity was noted with regard to carbohydrate digestion. *A. glabripennis* produces a large number of transcripts predicted to break down plant cell wall carbohydrates [31, 37], while in contrast, the gut community expressed numerous transcripts predicted to assimilate and ferment wood sugars (i.e., glucose and xylose). *A. glabripennis* also has the capacity to assimilate and use fermentation products via a diverse repertoire of insect-derived alcohol dehydrogenases [31].

Complementarity was also noted with regard to amino acid and vitamin metabolism. The microbiota expressed full pathways for the biosynthesis of branched chain and aromatic amino acids, while the beetle expressed full pathways for the decomposition of branched-chain amino acids, aromatic amino acids, and lysine [31], thus providing a direct example of how *A. glabripennnis* can directly catabolize nutrients synthesized by the gut microbial community. While the gut microbiota express transcripts involved in biosynthesis of methionine and vitamins, *A. glabripennis* itself expressed transcripts with predicted roles in recycling these essential nutrients. Further, the microbiome may augment production of non-essential amino acids, which may be important for nitrogen conservation in the nutrient poor environment of wood. For example, although *A. glabripennis* can synthesize cysteine, [31] the low abundance of sulfur in woody tissues may make cysteine a conditionally essential amino acid in this system. Therefore, the role of recycled sulfur obtained from taurine or other insect-derived compounds could also be critical for the synthesis of cysteine, methionine, and other sulfur-containing essential nutrients. The *A. glabripennis* transcriptome contains a larger repertoire of expressed peptidases and amino acid ligases relative to transcriptomes compiled from the guts of other phytophagous insects [31]. While the source of nitrogen used by larvae as they feed in the high C:N heartwood of their host trees is uncertain, this analysis suggests that the microbiota can provide a suitable store of amino acids and proteins that could be assimilated by *A. glabripennis* and its gut microbes.

### Potential contributions of microbial OTUs to *A. glabripennis*digestive physiology

In all beetle gut communities in this study we found rRNAs from several bacterial and fungal taxa that were taxonomically classified to the genera Fusarium, Pichia, and Pediococcus. Although these taxa all expressed transcripts that could serve key roles in the digestive and nutritional physiology of A. glabripennis, some of the potential contributions to metabolism were partitioned differently among taxa. For example, all three taxa expressed genes with predicted involvement in plant cell wall digestion (i.e. cellulases, β-glucosidases, β-xylosidases), nitrogen uptake and recycling, and detoxification. Transcripts with predicted involvement in xylose utilization were taxonomically assigned to Pediococcus and Pichia. However, several functions were differentially partitioned among these taxa. For example, the lactic acid bacterium Pediococcus had predicted roles in xylose utilization, sugar fermentation, aromatic amino acid biosynthesis, and vitamin biosynthesis, while genes involved in sterol biosynthesis were exclusively assigned to the fungal taxa.

#### Potential nutrition provisioning roles of Fusarium solani

OTUs taxonomically classified to the genus *Fusarium* are consistently found in the *A. glabripennis* larval midgut [20, 33] and are capable of secreting numerous plant cell wall degrading enzymes, detoxification enzymes, and laccases with putative involvement in lignin degradation [32]. Over 200 transcripts assigned to the genera *Fusarium* or *Nectria* (Family Nectriaceae) were detected in the gut contents library and many of these transcripts were classified as glycoside hydrolases (GH) based on Pfam domain annotations. One of the most striking findings was the presence of over 30 transcripts predicted to originate from GH 28 polygalacturonases, which have predicted involvement in degradation of pectin. While pectin is not prevalent in woody tissue, it is periodically deposited in secondary growth and represents stores of galactose sugars and essential minerals (e.g., calcium) [41] that could be assimilated by the beetle or gut fungi. A single transcript predicted to encode a GH 5 cellulase was also detected. While its ability to catalyze endo- or exo- type glucanase reactions could not be predicted based on *in silico* annotations alone, it could work in collaboration with beetle-derived cellulases to facilitate cell wall digestion [20, 27]. In addition, it was previously hypothesized that *F. solani* could play a key role in lignin degradation in the midgut [42]; several secreted laccases were detected previously through MudPIT shotgun proteomic analysis [32]. Although no known lignin peroxidase transcripts were detected in this analysis, *Fusarium*-derived transcripts with predicted involvement in metabolizing aromatic compounds were detected that could play accessory roles in processing the lignin biopolymer. For example, several transcripts were detected that are predicted to encode phenolic acid decarboxylases, aromatic ring hydroxylases, cytochrome P450s, and alcohol and aldehyde dehydrogenases, which can enhance oxidation of inter-phenylpropanoid linkages in lignin in the presence of other lignin degrading enzymes [43] or have roles in detoxification.

Non-entomopathogenic fungi associated with insects are also hypothesized to serve key roles in nutrient provisioning, including (but not limited to) sterol synthesis and nitrogen concentration [44]. Several transcripts derived from *Fusarium spp.* with predicted involvement in amino acid salvage and recycling of nitrogenous waste products were detected. These included nitrate transporters and xanthine/uracil family permeases, which can assimilate uric acid, xanthine, cysteine and other nitrogenous waste products. Additionally, transcripts linked to sterol biosynthetic pathways were detected and included transcripts with highest scoring BLASTX alignments to 3-keto sterol reductases, sulfatases, sterol regulatory element-binding protein (SREBP) cleavage-activating proteins, and di-trans-poly-cis-decaprenylcistransferases, which produce key intermediates in the biosynthesis of terpenoids.

#### Potential nutrition provisioning roles of yeasts in the A. glabripennis midgut

OTUs derived from yeasts are consistently detected in the midgut of *A. glabripennis* from field and laboratory reared larvae (unpublished data). The association of yeasts with beetle larvae has been extensively studied in a variety of cerambycids [7, 45, 46] and the importance of fungal enzymes to plant cell wall degradation in several cerambycid species has been demonstrated [47]. Previously, it was hypothesized that yeasts associated with the guts of multiple species of cerambycids ferment xylose [38] and the detection of transcripts derived from the xylose isomerase and various fermentation pathways provide support to this hypothesis. However, additional yeast-derived transcripts detected in this study suggest that these microbes could have roles in digestion and nutrient biosynthesis beyond xylose fermentation. Several yeast-derived transcripts annotated as multicopper oxidases, laccases, and C_α_ –dehydrogenases were detected. Although yeasts are not known to degrade lignin and laccases and C_α_ dehydrogenases are often involved in detoxification processes in yeasts, similar genes in other bacterial and fungal taxa can expedite oxidation of linkages in lignin, enhancing the degradation of lignin in the presence of other lignin degrading enzymes [48, 49]. To our knowledge, the ability of yeast laccases and C_α_ dehydrogenases to participate in lignin degradation has not been directly tested.

Yeasts associated with the beetle midgut also have the potential to produce enzymes with pivotal roles in nitrogen recycling. In addition to the glutamate dehydrogenase and taurine dioxygenase transcripts, which were derived predominately from yeasts, several transcripts annotated as putative uricases and ureases were detected, suggesting that yeasts associated with the midgut can also decompose and recycle nitrogenous waste products. Transcripts that encode essential nutrient biosynthetic pathways were also associated with yeasts, such as those involved in the synthesis of methionine, branched chain amino acids, aromatic amino acids, and α-ketoglutarate, suggesting that yeasts could also play roles in essential amino acid biosynthesis.

Several enzymes with predicted involvement in ergosterol biosynthesis were detected; it has been hypothesized that insects can utilize ergosterols produced by fungal symbionts for production of pheromones and cholesterol and, in some cases, biochemical evidence supports the utilization of fungal ergosterols [50]. Fungi associated with the midgut also produced transcripts that are predicted to encode enzymes involved in the biosynthesis of several vitamins, including riboflavin, thiamine, and thiazole, which are all deficient in woody tissue.

### Potential nutrient provisioning roles of lactic acid bacteria

By far, the most abundant microbial transcripts detected in the *A. glabripennis* midgut were taxonomically classified to the genus *Pediococcus* (Family Lactobacillaceae). This taxon has been previously detected in association with the *A. glabripennis* midgut [27] and lactic acid bacterial reads taxonomically classified to the genus *Leuconostoc* were previously detected through shotgun metagenomic and 16S rDNA amplicon analyses of beetles collected from another population of *A. glabripennis* [20]. Although no major pathways for carbohydrate digestion were associated with this taxon, pathways for the assimilation and utilization of β-1,4 linked di- and oligo-saccharides (e.g. cellobiose, β-1,4-linked xylose, arabinose, galactan, and rhamnose), N-acetylglucosamines (e.g., chitin and other aminosugars), and α-1,3 and α-1,6 linked mannose oligomers were highly expressed. This suggests that these bacteria metabolize and utilize the products of larger scale degradative processes catalyzed by either the beetle or other members of the gut community. The overabundance of β-glucosidases and cellobiose phosphotransferase systems associated with this genus and with the gut community in general can partially explain the enhanced cellulase complex activity in the presence of diverse gut microbial communities previously observed in the *A. glabripennis* midgut [27]. Heightened β-glucosidase activities could reduce the impact of end product inhibition of cellulases. Aldose epimerases were also detected in association with this taxon, which can catalyze the inter-conversion of α-D-glucose and β-D-glucose. This may explain the overabundance of α-glucoside transporters in the *A. glabripennis* midgut transcriptome [31] relative to the microbial community since the beetle may acquire some of its carbon sources from α-D-glucose synthesized by various members of the midgut community. Aldo-keto reductases (AKR) and laccases associated with the genus *Pediococcus* were also highly abundant. AKRs were highly abundant in both the *A. glabripennis* larval midgut transcriptome [31] and larval midgut metagenome [20] where they were hypothesized to serve key roles in lignin degradation because they can enhance the cleavage of β-aryl ether linkages in the presence of other lignin degrading enzymes. Aside from lignin degradation, aldo-keto reductases can be integrally involved in detoxification, metabolism of monosaccharides, and a variety of other diverse oxidoreductive processes; thus, the roles of these transcripts in the midgut could be diverse. Finally, many transcripts were detected with predicted roles in the biosynthesis of vitamins, including folate, coenzyme A, and thiamine in the biosynthesis of branched chain amino acids, lysine, asparagine, arginine, aspartate, and aromatic amino acids.

Previous analysis of metagenomic DNA predicted to originate from bacteria assigned to the genus *Leuconostoc* detected in *A. glabripennis* midguts collected from an established population in Worcester, MA indicated that these lactic acid bacteria have a similar metabolic potential as the *Pediococcus*-derived transcripts detected in the current study [20]. Currently, the relationship between *A. glabripennis* and these lactic acid bacteria is uncharacterized; however, the conversion of xylose sugars released by insect-derived xylanase and the synthesis of essential amino acids and other nutrients that are generally deficient from woody tissue suggests that these bacteria could be beneficial.

## Conclusions

The midgut microbiome of *A. glabripennis* is enriched in biosynthetic pathways for the synthesis of essential amino acids, vitamins, and sterols. Consequently, microbes can provide *A. glabripennis* with essential nutrients that the beetle cannot obtain directly from its woody diet, or synthesize itself. The gut microbiota also produces its own suite of transcripts that can enhance lignin degradation, degrade hemicellulose, and ferment xylose and wood sugars. An abundance of cellulases (from several glycoside hydrolase families) plus transcripts that allow the beetle to convert microbe-synthesized essential amino acids into non-essential amino acids are expressed by the beetle. The beetle and its gut microbiota likely collaborate to digest carbohydrates and convert released sugars and amino acid intermediates into essential nutrients otherwise lacking from woody plants. Further, the nutritional provisioning capabilities of the *A. glabripennis* gut microbiome may contribute to the unusually broad host range of this invasive beetle species.

Many studies have sought to characterize the microbiota associated with xylophagous insects to determine how they are able to thrive in woody tissue and to help develop targets for biological control. The challenge is that these communities are taxonomically diverse, temporally dynamic and composed of large numbers of facultative microbial taxa, fueling debate about whether such microbes enhance fitness or are transient commensals [7, 8, 51]. The presence of some of the same microbial taxa in the midguts of *A. glabripennis* and exemplars of other beetle lineages that contain large numbers of tree-feeding species (e.g., the Buprestidae [8] and Scolytinae), in addition to the apparent importance of certain OTUs (e.g., *Fusarium solani* and yeasts) to the nutritional ecology of *A. glabripennis* and other wood-feeding beetles (e.g., (Scarabaeidae) [52]; (Passalidae) [38]; (Curculionidae) [53]), suggests that indepedently-derived partnerships with microbes may be common facilitators of taxonomic and ecological diversification in beetles, as they are in certain other groups of insects (e.g., aphids [11]). These partnerships likely facilitate access to novel food sources through enhanced nutritional provisioning capabilities. Interestingly, several expressed rRNAs and mRNAs predicted to originate from rare OTUs were also detected in this study, indicating that persistence and abundance are not necessarily correlated with transcriptional activity and that even rare microbial taxa can express genes integral to nutritional ecology.

Microbes associated with xylophagous insects have been hypothesized to have primary involvement in the degradation of woody tissue; however, our analyses demonstrate that pathways for the synthesis of essential nutrients, including aromatic and branched chain amino acids, sterols, and vitamins, and for recycling of nitrogenous waste products, are relatively more abundant, suggesting that the majority of the microbial community may be more important for nutrient provisioning. In addition, the *A. glabripennis* microbiota produce transcripts with predicted roles in pentose sugar fermentation and utilization, which could allow xylose to be used as a substrate for energy production and essential and non-essential amino acid biosynthesis.

Although no canonical lignin degrading genes were detected, several enzymes with axillary roles in this process were identified, including C_α_ dehydrogenases, multicopper oxidases, laccases, and aldo-keto reductases, suggesting that the gut microbiota can facilitate the degradation of lignin [42]. In contrast, *A. glabripennis* produces several endogenous cellulases, xylanases, β-glucosidases, and other cell wall degrading enzymes, suggesting that the beetle has a primary role in digesting plant polysaccharides and its interactions with microbes could augment these processes. Therefore, disrupting microbes with predicted involvement in essential nutrient biosynthesis, such as lactic acid bacteria and yeasts, could have deleterious phenotypic impacts on *A. glabripennis* and serve as a viable approach for controlling populations of *A. glabripennis* and other wood-feeding beetles.

Many of the same bacterial genera detected in the *A. glabripennis* midgut are shared among all life stages of the phloephagous emerald ash borer (Buprestidae: *Agrilus planipennis*) [8]. *Agrilus planipennis* and *A. glabripennis* have different biologies (feeding primarily in phloem and xylem, respectively) and are very distantly related (last common ancestor ~200 MYA) [54], suggesting that their wood-feeding habits evolved independently. Similarly, the bark beetle *Dendroctonus ponderosae,* which last shared a common ancestor with *A. glabripennis* ~170 MYA [55], produces different types of cell wall degrading enzymes than *A. glabripennis*, suggesting that these beetles, while members of sister superfamilies, also likely have independently evolved to feed in trees*.* No well-resolved and/or comprehensive higher-level molecular phylogenies are available for Cerambycidae or their closest relatives and few data are available about their gut microbiota, making it difficult to speculate on the timing and number of phylogenetic origins of xylophagy in longhorned beetles or in Chrysomeloidea as a whole. Nonetheless, through multiple comparisons of microbial communities associated with xylophagous beetles, it should be possible to characterize similarities and differences in microbial community composition and function and genetic/physiological adaptations associated with xylophagy within the species rich family Cerambycidae, and also within other families of Coleoptera with xylophagous members.

While this study demonstrates that various members of the gut microbiome express genes that can complement the beetle’s existing repertoire of digestive and nutrient scavenging capabilities, more research is needed to determine which of these symbionts are required for survival in woody tissue. However, experiments to manipulate the composition of the gut community in *A. glabripennis* and other phloephagous and xylophagous beetles to demonstrate the impact of disruption of select microbes on fitness and digestive physiology have been impeded by the difficulty of rearing these insects on anti-microbial free artificial diet. Also, the composition of the gut microbial community and the genes expressed by its members were sampled only at a single life stage in this study (third instar) and therefore, this analysis cannot account for shifts in community composition and its metabolic potential that may occur as the larvae feed in different tissues of the tree throughout their life cycle.

Rarefaction curves computed for the 16S bacterial communities were not completely saturated, indicating that OTUs present in lower relative abundances in the midgut may not have been sampled for sequencing. Although deeper sequencing and a more comprehensive survey of the microbial genes expressed in the gut would allow us to identify additional microbial contributions to digestive physiology and more opportunities for nutrient exchange, it is often difficult to achieve complete saturation in complex communities with large numbers of rare OTUs as has been previously observed in *A. glabripennis* [17, 27, 31] and other wood-feeding insects [56]. Both the community and the expression of various digestive and nutrient scavenging genes are dynamic; therefore, this survey is not exhaustive and additional bacterial and fungal OTUs and microbial contributions to digestive physiology will likely be identified with further study.

## Methods

### Fungal and bacterial communities of the *A. glabripennis*midgut

We characterized the bacterial and fungal communities associated with the *A. glabripennis* midgut by high-throughput sequencing of 16S bacterial rRNA and fungal ITS amplicon libraries. Larval *A. glabripennis* were reared on living, potted *Acer saccharum* (sugar maple) in a USDA-approved quarantine greenhouse as described previously [27, 31]. *A. glabripennis* adults and larvae used in these experiments were reared in colony at The Pennsylvania State University. This colony is of mixed ancestry from several invasive populations of *A. glabripennis* obtained within the U.S. and has been in culture for over 10 years, but field-collected insects from regulated areas in the U.S. are routinely added to the colony to maintain genetic diversity. In brief, potted *A. saccharum* trees were grown at an outdoor nursery until they were 3–4 years old. Several weeks before use, trees were moved into the quarantine greenhouse to allow for acclimation to greenhouse conditions. Three trees were placed in a walk-in insect cage (~3 m high, 3 m long, and 2 m wide) and five mating pairs of *A. glabripennis* adults were placed in the cage and allowed to mate and lay eggs.

After 8 weeks of feeding in trees, larvae were removed and surface sterilized with two washes of 70% ethanol followed by a single wash in sterile distilled water to remove residual ethanol. Larvae were dissected and their midguts removed for DNA extraction using the PowerSoil DNA Isolation Kit (MoBio, Carlsbad, CA). DNA integrity and concentration were verified using a NanoDrop spectrophotometer (Thermo-Fisher, Pittsburgh, PA) and the Quant-It dsDNA assay (Life Technologies, Carlsbad, CA), which was analyzed on a Qubit fluorometer (Life Technologies). DNA collected from each midgut was used to construct partial 16S bacterial amplicon libraries, ranging from position 27 F to 907R, and full ITS amplicon libraries, ranging from ITS5 to ITS4. 454 multiplex identifiers (MIDs) and 454 Titanium library adapters were directly incorporated into the primer sequences as described previously [20]. In brief, 100 ng of DNA were added to a PCR reaction mixture containing 1.0 μL 10X Buffer Mix (Roche, Branford, CT), 2 mM dNTPs (Roche), 0.5 U Taq polymerase (Roche), 5 μM forward primer (27 F: 5’-AGAGTTTGATCMTGGCTCAG-3’) and 5 μM reverse primer (907R: 5’-CCCCGTCAATTCMTTTGAGTTT-3’) [57]. PCR cycling conditions were as follows: initial denaturation for 3 minutes at 94°C, 30 cycles of 94°C for 15 seconds, 55°C for 45 seconds, and 72°C for 1 minute, and a final extension at 72°C for 8 minutes. The PCR reaction mixture and thermal cycling conditions for the ITS amplicon library were identical to those used to generate the bacterial library except that the 5 μM forward primer (ITS5: 5’-GGAAGTAAAAGTCGTAACAAGG-3’) and the 5 μM reverse primer (ITS4: 5’- TCCTCCGCTTATTGATATGC-3’) [58] were substituted and 35 PCR cycles were used. PCR products were evaluated using agarose gel electrophoresis and bands corresponding to the sizes of the desired products were eluted from the gel using the Agarose Gel Extraction Kit (Roche). For the 16S amplicon libraries, products of approximately 900 bp in size were eluted from the gel. Since the length of the ITS region varies in different fungal taxa, PCR products ranging in size from 500 bp to 1800 bp were eluted from the gel and were used for library preparation. Libraries were quantified using the Qubit dsDNA assay (Invitrogen), samples were multiplexed, and library titers were calculated using quantitative PCR against a library standard (Kapa Biosystems, Woburn, MA). Approximately 5,000 reads were sequenced from each 16S bacterial library and 1,000 reads were sequenced from each ITS fungal library using 454 Titanium XLR chemistry. Raw reads from each 16S and ITS library were deposited in the NCBI Sequence Read Archive (SRA) under the accession numbers SRX367813 and SRX369139, respectively. These reads are associated with BioProjects PRJNA222386 and PRJNA222384, respectively.

### Operational taxonomic unit-based analysis of microbial 16S and ITS amplicons from the *A. glabripennis*midgut

16S amplicons were assigned to OTUs using the program Mothur (version 1.32.0). Pyrosequencing flowgrams were denoised to reduce the impact of 454 homopolymer errors on OTU categorization [59, 60] and low quality amplicons with ≥ 80% of the bases containing a quality score of less than 25 were removed from the dataset. Chimeras were detected and removed with the program UCHIME [61], high quality reads greater than 700 bp in length were clustered into OTUs at 97% similarity; rarefaction curves, richness estimates, and other indices of ecological diversity were computed using Mothur [62]. Bacterial OTUs were classified to higher taxa using the Ribosomal Database Project (RDP) Classifier [63], with an 80% confidence threshold for taxonomic classifications [60]; sequences classified as mitochondrial, chloroplast, or eukaryotic in origin were omitted from the analysis. Before calculating richness and diversity metrics, the 16S and ITS amplicon libraries were normalized by randomly subsampling (without replacment) the same number of reads from each library to equalize sampling depth across communities.

For analysis of ITS amplicons, flowgrams were denoised and low quality reads discarded as described above. High quality reads ranging from 450 bp to 850 bp in length were clustered into OTUs at 97% sequence similarity. However, ITS is highly prone to indel events, making it difficult to accurately align across distantly related taxa [64], leading to artificial inflations in richness calculations and estimates when using alignment-based methods for OTU classification [65]. Therefore, instead of implementing Mothur’s alignment based approach for OTU assignment, amplicons were clustered into OTUs at 97% sequence similarity using the program CD-HIT-EST [66]. The program UCHIME [61] was used to detect putative chimeras using the more highly abundant OTUs as a reference, since templates for chimera detection were not available for the ITS region. Taxonomic classification of ITS OTUs was conducted by first comparing representative sequences from each OTU to NCBI’s non-redundant nucleotide database using BLASTN [67] to identify plant or insect derived ITS amplicons. After excluding these, fungal OTUs were classified to higher taxa using the UNITE database [68] at a 90% confidence level and diversity and richness indices were computed using Mothur. A higher confidence threshold was used for fungal classification to ensure accurate taxonomic assignment despite the relative underrepresentation of fungal taxa in NCBI databases. As in the 16S analysis, the ITS amplicon libraries were normalized by randomly subsampling the same number of reads from each library before computing and comparing diversity indices. To determine which OTUs were transcriptionally active in the midgut, rRNAs and fungal ITS sequences assembled from the Illumina data were also compared to representative sequences derived from each 16S or ITS OTU detected through amplicon sequencing via BLASTN. Due to potential homopolymer errors in OTU sequences derived from 454 amplicons, rRNA and ITS sequences from the transcriptome data were considered to have a significant match if they were ≥ 95% identical at the nucleotide level to an OTU detected through 454 amplicon sequencing.

### Metatranscriptome sequencing of the *A. glabripennis*midgut

Larvae feeding in *A. saccharum* for 8 weeks were removed from the tree and midguts collected from 10 instars. RNA was sampled from two gut locations. First, total RNA was sampled from the midgut contents collected from five larvae to identify genes from transcriptionally active microbes associated with the food bolus. Second, total RNA was sampled from intact midguts removed from four larvae to capture RNAs expressed by microbes that may be associated with the gut tissue. This is of significant interest because microbes are often observed in the epithelium of the *A. glabripennis* midgut and are poised to directly interact with host cells. One shortfall of the previous metagenomic survey of the *A. glabripennis* midgut microbiota [20] was that only microbes associated with the midgut contents were sampled and sequenced, and microbes associated with the midgut epithelial cells could have been missed.

### Shotgun sequencing of mRNA from *A. glabripennis*midgut contents

Third instars of *A. glabripennis* were reared in potted *A. saccharum*, then collected and dissected under sterile conditions as described above, except in this case the peritrophic matrix was removed to enrich the sample for microbial cells associated with the food bolus. Midgut contents were flash frozen in liquid nitrogen and total RNA was immediately extracted using the FastRNA Spin Kit for Soil (MP Biomedicals, Solon, OH). A post-RNA extraction clean-up was performed using RNA Clean & Concentrator (Zymo Research, Irvine, CA) to remove residual salts and phenolics; the sample was also treated with DNase I (Zymo Research, Irvine, CA). Sample integrity was verified using the RNA Pico Assay (Life Technologies, Carlsbad, CA) and Nano Drop (Thermo-Fisher), while the sample concentration was determined with the Quant-It RNA Assay (Life Technologies). Removal of DNA was confirmed using the Quant-iT High-Sensitivity DNA Assay (Life Technologies, Carlsbad, CA). Insect- and microbe-derived rRNAs were depleted from the sample as described previously [31].

The total RNA recovered from the midgut contents was of relatively low quality (RNA integrity score of 5, on a scale ranging from 1 to 10) and the amount of recovered RNA was low due to the presence of nucleases and harsh conditions within the midgut lumen. To obtain sufficient RNA for sequencing, 20 ng of enriched mRNA was amplified using Ovation RNA Seq (NuGEN, San Carlos, CA) to produce 2 μg of double-stranded cDNA. The library was sheared using a Covaris Focused-ultrasonicator (Covaris, Woburn, MA), enriched for 175 nt fragments, and prepared using TruSeq Genomic DNA library adapters (Illumina, San Diego, CA). Approximately 40 million 130 nt paired end reads (a total of 10.6 Gb sequence data) were obtained using the Illumina GAIIx platform. An overlapping paired end library enriched for 175 nt fragments was constructed so that a longer contiguous sequence of this length could be constructed by merging a single read pair, thus reducing the likelihood of cross-assembling reads derived from orthologous genes from different microbial taxa [69]. The raw Illumina reads were deposited in NCBI’s SRA under the accession number SRX352195 and are associated with Bioproject PRJNA219402.

### Shotgun sequencing of mRNA collected from *A. glabripennis*midgut tissue

Four individual third instars of *A. glabripennis* were dissected and their midguts removed, pooled, total RNA extracted, and ribosomal RNA depleted, as described above. The quality and concentration of RNA recovered from the intact midguts were high relative to the above-mentioned gut contents library, so amplification prior to library construction was not necessary. Approximately 200 ng of enriched RNA was used for library preparation with the TruSeq RNA Library Prep Kit (Illumina), omitting the polyA enrichment step to enhance recovery of non-polyA bacterial mRNA. The library was enriched for 175 nt fragments so that paired end reads overlapped by ~30 nt. Approximately 130 million 101 nt paired end reads (36 Gb) were generated using the Illumina HiSeq 2000 platform. Raw reads were deposited in NCBI’s SRA under the accession number SRX265389 and are associated with Bioproject PRJNA193436.

### Assembly of the *A. glabripennis*larval midgut metatranscriptome

Due to differences in library construction between the whole gut and gut contents libraries, the two libraries were quality filtered and assembled separately. Low quality reads or reads with ≥ 20% of the bases possessing quality scores less than 20 were filtered from the dataset using the FastX Toolkit and residual library adapters were removed using Cutadapt [70]. Remaining read pairs and orphans were assembled with the Trinity *de novo* assembler [71] in paired-end mode. To reduce the coverage of highly expressed genes and to improve the ability to assemble transcripts and transcript isoforms originating from lowly expressed genes, k-mers (k = 25) from quality filtered Illumina paired end reads were reduced to ≤ 30X coverage using digital normalization [72]. Normalized reads were assembled with Trinity (version r2012-10-05) [71]. We used Trinity for assembly due to its ability to discriminate and assemble gene isoforms and splice variants in data from Eukaryotes [71]. We anticipated that Trinity’s ability to distinguish between isoforms from the same gene would prevent cross-assembling reads originating from orthologous genes from different microbial taxa and/or strains.

### Annotation of metatranscriptome genes and isoforms

Transcripts assembled by Trinity from both the midgut tissue and midgut contents libraries were used in downstream annotations and analyses. Since transcript isoforms could be derived from orthologous genes from closely related bacterial species or strains, they were not collapsed to the gene level prior to *in silico* functional annotation. SSU (small subunit) and LSU (large submit) rRNAs were detected and removed with HMMer [73] using profiles for prokaryotic, eukaryotic, and archeael SSU, LSU, and 5.8S/8S rRNAs [74]. Bacterial, fungal, insect, and tree SSU and LSU rRNAs detected in the transcriptome were taxonomically classified by comparison to the Silva database, a manually curated collection of high-quality, full length SSU and LSU ribosomal RNAs from all domains of life [75]. The remainder of the isoforms were annotated by a BLASTX comparison [67] to the NCBI non-redundant protein database and were taxonomically classified using MEGAN metagenomic analyzer [76] to identify transcripts that were likely microbial in origin. Microbial transcripts were functionally grouped into Gene Ontology terms [77] and mapped onto KEGG pathways [78] using the Trinotate pipeline and the KAAS server [79], respectively. BLASTX results were corroborated and glycoside hydrolase (GH) family assignments were computed by scanning for Pfam A domains [80] using HmmSearch [81]. Transcripts were also aligned to the *A. glabripennis* genome using BLASTN to ensure that they did not arise from genes that were laterally transferred from microbes to *A. glabripennis*.

### Comparison of microbial and insect annotations

To determine how the gut microbiota can augment and complement *A. glabripennis’* endogenous digestive repertoire, annotations of microbial genes assembled from the intact midgut and midgut contents libraries were compared to annotations of insect-derived genes that were previously assembled and annotated from the *A. glabripennis* midgut [33]. The full genome sequence of *A. glabripennis* is available pre-release and was used to confirm the absence of various host digestive genes and metabolic pathways.

### Availability of supporting data

Raw reads from each 16S and ITS library were deposited in the NCBI Sequence Read Archive (SRA) under the accession numbers SRX367813 and SRX369139, respectively. These reads are associated with BioProjects PRJNA222386 and PRJNA222384, respectively. Barcoding information is presented in Additional file 5: Table S4. Raw Illumina reads from the whole midgut library were deposited in NCBI’s SRA under the accession number SRX265389 and are associated with Bioproject PRJNA193436. The raw Illumina reads for the midgut contents library were deposited in NCBI’s SRA under the accession number SRX352195 and are associated with Bioproject PRJNA219402. The assemblies and annotations for the *A. glabripennis* midgut transcriptome used for comparisons in this manuscript can be found in in NCBI’s Transcript Shotgun Assembly database under the accession number [GALX00000000] and was previously published [28]. Assembled microbial transcripts from the intact midgut and midgut contents and their corresponding annotations are included as Additional file 6: Supplemental file 1 and Additional file 7: Supplemental file 2. Highest scoring BLASTX results for the intact midgut and midgut contents are presented in Additional file 8: Supplemental file 3 and Additional file 9: Supplemental file 4.

## Electronic supplementary material

Additional file 1: Table S1: Taxonomic classifications of 16S bacterial OTUs detected in all A. glabripennis larval midguts sampled. (DOCX 18 KB)

Additional file 2: Figure S1: Taxonomic composition of midgut contents (top) and intact midgut (bottom) metatranscriptome libraries. (TIF 1 MB)

Additional file 3: Table S2: Taxonomic classification of microbial rRNAs detected in the midgut contents and intact midgut libraries. (DOCX 19 KB)

Additional file 4: Table S3: Number of unique KO terms found in KEGG pathways associated with carbon metabolism, nitrogen acquisition and amino acid biosynthesis, nutrient acquisition, and detoxification. (DOCX 21 KB)

Additional file 5: Table S4: 454 Barcodes used for 16S and ITS amplicon studies. (DOCX 11 KB)

Additional file 6: **Supplemental file 1.** Fasta file containing microbial transcripts from the A. glabripennis intact midgut. (ZIP 823 KB)

Additional file 7: **Supplemental file 2.** Fasta file containing microbial transcripts assembled from the A. glabripennis intact midgut. (ZIP 419 KB)

Additional file 8: **Supplemental file 3.** Highest scoring BLASTX alignments of protein coding genes assembled from the A. glabripennis midgut contents. (XLSX 251 KB)

Additional file 9: **Supplemental file 4.** Highest scoring BLASTX alignments of protein coding genes assembled from the A. glabripennis intact midgut. (XLSX 89 KB)
