# Supplementary material for: Functional genomics and microbiome profiling of the Asian longhorned beetle (Anoplophora glabripennis) reveal insights into the digestive physiology and nutritional ecology of wood feeding beetles
Source: BMC Genomics. 2014 Dec 12;15(1):1096. doi: 10.1186/1471-2164-15-1096 (PMC4299006; doi:10.1186/1471-2164-15-1096)
Supplement: Supplementary file 1 — Additional file 1: Table S1: Taxonomic classifications of 16S bacterial OTUs detected in all A. glabripennis larval midguts sampled. (DOCX 18 KB) [file 12864_2014_6803_MOESM1_ESM.docx]

| Phylum | Class | Family | Number of OTUs |
| --- | --- | --- | --- |
| Actinobacteria | Actinobacteria | Propionibacteriaceae | 1 |
|  |  | Propionibacteriaceae | 1 |
| Bacteroidetes | Sphingobacteria | Chitinophagaceae | 1 |
|  |  | Prevotellaceae | 1 |
|  |  | Sphingobacteriaceae | 1 |
| Firmicutes | Bacilli | Staphylococcaceae | 1 |
|  |  | Streptococcaceae | 3 |
| Proteobacteria | Alphaproteobacteria | Brucellaceae | 1 |
|  |  | Methylocystaceae | 1 |
|  |  | Sphingomonadaceae | 1 |
|  | Betaproteobacteria | Burkholderiaceae | 1 |
|  |  | Comamonadaceae | 1 |
|  | Gammaproteobacteria | Enterobacteriaceae | 3 |
|  |  | Pasteurellaceae | 1 |
|  |  | Pseudomonadaceae | 1 |
|  |  | Xanthomonadaceae | 2 |
