# Supplementary figures and images for: Functional genomics and microbiome profiling of the Asian longhorned beetle (Anoplophora glabripennis) reveal insights into the digestive physiology and nutritional ecology of wood feeding beetles
Source: BMC Genomics. 2014 Dec 12;15(1):1096. doi: 10.1186/1471-2164-15-1096 (PMC4299006; doi:10.1186/1471-2164-15-1096)

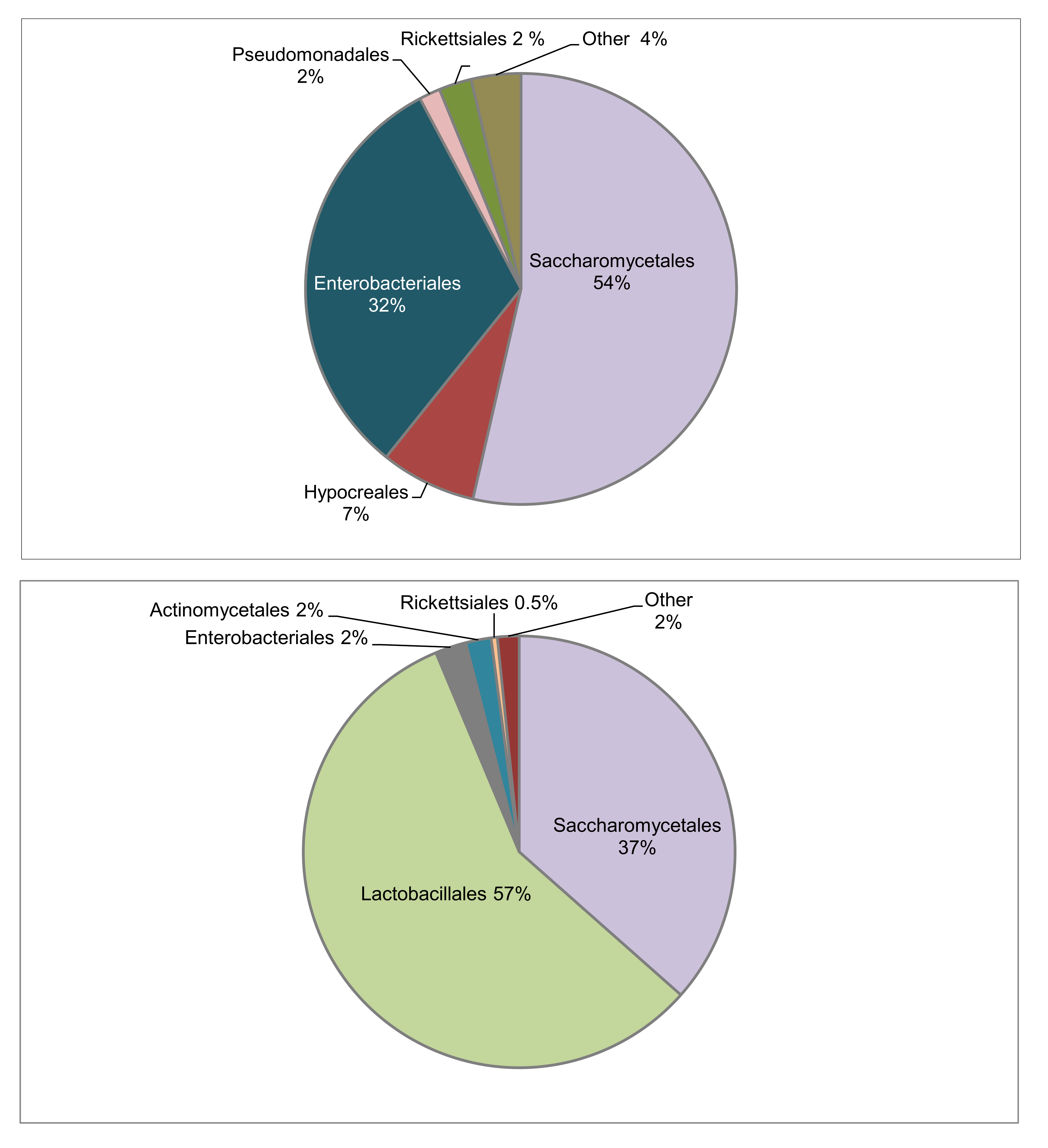

Supplement: Supplementary file 2 — Additional file 2: Figure S1: Taxonomic composition of midgut contents (top) and intact midgut (bottom) metatranscriptome libraries. (TIF 1 MB) [file 12864_2014_6803_MOESM2_ESM.tif]
