# Supplementary material for: Functional genomics and microbiome profiling of the Asian longhorned beetle (Anoplophora glabripennis) reveal insights into the digestive physiology and nutritional ecology of wood feeding beetles
Source: BMC Genomics. 2014 Dec 12;15(1):1096. doi: 10.1186/1471-2164-15-1096 (PMC4299006; doi:10.1186/1471-2164-15-1096)
Supplement: Supplementary file 3 — Additional file 3: Table S2: Taxonomic classification of microbial rRNAs detected in the midgut contents and intact midgut libraries. (DOCX 19 KB) [file 12864_2014_6803_MOESM3_ESM.docx]

| **Kingdom** | **Phylum** | **Class** | **Order** | **Number Unique Transcripts** | **Type** |
| --- | --- | --- | --- | --- | --- |
| Bacteria | Actinobacteria | Actinobacteria | Actinomycetales | 105 | 16S, 23S |
|  | Bacteroidetes | Unclassified Bacteroidetes | Unclassified | 4 | 23S |
|  |  | Sphingobacteria | Sphingobacteriales | 11 | 16S, 23S |
|  | Deinococcus-Thermus | Deinococci | Deinococcales | 5 | 23S |
|  | Firmicutes | Bacilli | Bacillales | 15 | 16S, 23S |
|  |  |  | Lactobacillales | 14 | 16S, 23S |
|  |  |  | Unclassified Bacilli | 7 | 16S, 23S |
|  | Proteobacteria | Alphaproteobacteria | Rhizobiales | 12 | 16S, 23S |
|  |  |  | Rickettsiales | 6 | 23S |
|  |  |  | Sphingomonadales | 16 | 16S, 23S |
|  |  |  | Unclassified Alphaproteobacteria | 3 | 23S |
|  |  | Betaproteobacteria | Burkholderiales | 24 | 16S, 23S |
|  |  |  | Unclassified Betaproteobacteria | 4 | 23S |
|  |  | Gammaproteobacteria | Enterobacteriales | 29 | 16S,23S |
|  |  |  | Pseudomonadales | 19 | 16S, 23S |
|  |  |  | Unclassified Gammaproteobacteria | 4 | 23S |
|  |  | Unclassified Proteobacteria | Unclassified | 8 | 23S |
|  | Unclassified Bacteria | Unclassified | Unclassified | 7 | 16S, 23S |
| Fungi | Ascomycota | Saccharomycetes | Saccharomycetales | 48 | 18S, 28S |
|  |  | Sodariomycetes | Hypocreales | 24 | 8S, 18S, 28S |
|  |  |  | Unclassified Sodariomycetes | 6 | 28S |
|  | Basidiomycota | Unclassified Basidiomycota | Unclassified | 7 |  |
|  | Unclassified Fungi | Unclassified | Unclassified | 9 | 8S, 18S, 28S |
