# Supplementary material for: Functional genomics and microbiome profiling of the Asian longhorned beetle (Anoplophora glabripennis) reveal insights into the digestive physiology and nutritional ecology of wood feeding beetles
Source: BMC Genomics. 2014 Dec 12;15(1):1096. doi: 10.1186/1471-2164-15-1096 (PMC4299006; doi:10.1186/1471-2164-15-1096)
Supplement: Supplementary file 4 — Additional file 4: Table S3: Number of unique KO terms found in KEGG pathways associated with carbon metabolism, nitrogen acquisition and amino acid biosynthesis, nutrient acquisition, and detoxification. (DOCX 21 KB) [file 12864_2014_6803_MOESM4_ESM.docx]

|  | ***Anoplophora glabripennis*** | **Gut Microbes** |
| --- | --- | --- |
|  |  |  |
| **Carbon Metabolism Pathways** | | |
| 2-Oxocarboxylic acid metabolism | 7 | 18 |
| ABC transporters | 8 | 36 |
| Amino sugar and nucleotide sugar metabolism | 22 | 30 |
| Butanoate metabolism | 11 | 12 |
| C5-Branched dibasic acid metabolism | 0 | 4 |
| Carbon Metabolism | 36 | 67 |
| Citrate cycle TCA cycle | 14 | 22 |
| Fructose and mannose metabolism | 11 | 19 |
| Galactose metabolism | 11 | 16 |
| Glycolysis / Gluconeogenesis | 22 | 28 |
| Pentose and glucuronate interconversions | 12 | 13 |
| Pentose phosphate pathway | 7 | 18 |
| Phosphotransferase system PTS | 1 | 13 |
| Propanoate metabolism | 13 | 15 |
| Pyruvate metabolism | 13 | 31 |
| Starch and sucrose metabolism | 17 | 23 |
| **Nitrogen acquisition and amino acids synthesis** | | |
| Alanine, aspartate and glutamate metabolism | 20 | 21 |
| Arginine and proline metabolism | 20 | 19 |
| beta-Alanine metabolism | 14 | 6 |
| Biosynthesis of amino acids | 32 | 67 |
| Cyanoamino acid metabolism | 2 | 4 |
| Cysteine and methionine metabolism | 15 | 17 |
| D-Alanine metabolism | 0 | 3 |
| D-Glutamine and D-glutamate metabolism | 1 | 2 |
| Glutathione metabolism | 14 | 10 |
| Glycine, serine and threonine metabolism | 16 | 17 |
| Histidine metabolism | 6 | 3 |
| Lysine biosynthesis | 3 | 8 |
| Lysine degradation | 13 | 5 |
| Nitrogen metabolism | 4 | 9 |
| Phenylalanine metabolism | 7 | 6 |
| Phenylalanine, tyrosine and tryptophan biosynthesis | 3 | 9 |
| Purine metabolism | 54 | 61 |
| Pyrimidine metabolism | 42 | 49 |
| Selenocompound metabolism | 4 | 6 |
| Taurine and hypotaurine metabolism | 3 | 6 |
| Tryptophan metabolism | 8 | 8 |
| Tyrosine metabolism | 10 | 10 |
| Valine, leucine and isoleucine biosynthesis | 1 | 6 |
| Valine, leucine and isoleucine degradation | 22 | 7 |
| **Nutrient Acquisition** | |  |
| Biosynthesis of unsaturated fatty acids | 4 | 5 |
| Biotin metabolism | 3 | 4 |
| Fatty acid biosynthesis | 5 | 9 |
| Fatty acid degradation | 19 | 8 |
| Fatty acid elongation | 7 | 1 |
| Folate biosynthesis | 5 | 4 |
| Insect hormone biosynthesis | 3 | 0 |
| Lipoic acid metabolism | 2 | 1 |
| Nicotinate and nicotinamide metabolism | 8 | 6 |
| One carbon pool by folate | 5 | 12 |
| Pantothenate and CoA biosynthesis | 7 | 7 |
| Riboflavin metabolism | 5 | 6 |
| Steroid biosynthesis | 4 | 7 |
| Steroid hormone biosynthesis | 4 | 0 |
| Sulfur metabolism | 4 | 7 |
| Terpenoid backbone biosynthesis | 9 | 11 |
| Thiamine metabolism | 3 | 9 |
| Ubiquinone and other terpenoid-quinone biosynthesis | 3 | 3 |
| Vitamin B6 metabolism | 4 | 3 |
| **Detoxification** | |  |
| Aminobenzoate degradation | 6 | 3 |
| Atrazine degradation | 0 | 2 |
| Benzoate degradation | 3 | 4 |
| Bisphenol degradation | 1 | 0 |
| Caprolactam degradation | 4 | 2 |
| Chloroalkane and chloroalkene degradation | 4 | 3 |
| Dioxin degradation | 0 | 2 |
| Drug metabolism - cytochrome P450 | 7 | 3 |
| Drug metabolism - other enzymes | 15 | 5 |
| Ethylbenzene degradation | 0 | 2 |
| Metabolism of xenobiotics by cytochrome P450 | 8 | 3 |
| Naphthalene degradation | 3 | 4 |
| Nitrotoluene degradation | 1 | 0 |
| Polycyclic aromatic hydrocarbon degradation | 2 | 2 |
| Styrene degradation | 1 | 0 |
| Toluene degradation | 0 | 4 |
| Xylene degradation | 0 | 2 |
