# Supplementary material for: Functional genomics and microbiome profiling of the Asian longhorned beetle (Anoplophora glabripennis) reveal insights into the digestive physiology and nutritional ecology of wood feeding beetles
Source: BMC Genomics. 2014 Dec 12;15(1):1096. doi: 10.1186/1471-2164-15-1096 (PMC4299006; doi:10.1186/1471-2164-15-1096)
Supplement: Supplementary file 5 — Additional file 5: Table S4: 454 Barcodes used for 16S and ITS amplicon studies. (DOCX 11 KB) [file 12864_2014_6803_MOESM5_ESM.docx]

**Table S4. 454 Barcodes used for 16S and ITS amplicon studies.**

| **Sample ID** | **16S barcode** | **ITS barcode** |
| --- | --- | --- |
| SM10 | ACGAGTGCGT | AGAGCGTCAC |
| SM11 | ACGCTCGACA | AGCGACTAGC |
| SM12 | AGACGCACTC | AGTGACACAC |
| SM13 | AGCACTGTAG | TCGCTGCGTA |
